# Association between Altered Expression and Genetic Variations of Transforming Growth Factor β-Smad Pathway with Chronic Myeloid Leukemia

**Published:** 2018-01-01

**Authors:** Yogender Shokeen, Neeta Raj Sharma, Abhishek Vats, Veronique Dinand, Mirza Adil Beg, Satish Sanskaran, Sachin Minhas, Mayank Jauhri, Arun K Hariharan, Vibha Taneja, Shyam Aggarwal

**Affiliations:** 1 Department of Medical Oncology, Sir Ganga Ram Hospital, Delhi, India; 2 Department of Research, Sir Ganga Ram Hospital, Rajinder Nagar, Delhi, India; 3 School of Biotechnology and Biosciences, Lovely Professional University, Jalandhar, Punjab, India; 4 Strand Center for Genomics and Personalized Medicine, UAS Alumni Building, Veterinary College Campus, Bellary Road, Hebbal, Bangalore, India

**Keywords:** Chronic myeloid leukemia (CML), SMAD, SMAD7, TGFβ-Smad pathway, TGFβ1, TGFβR1, TGFβR2

## Abstract

**Background:** Chronic myeloid leukemia (CML) is a hematological disorder caused by fusion of *BCR* and *ABL* genes. *BCR-ABL* dependent and independent pathways play equally important role in CML. TGFβ-Smad pathway, an important *BCR -ABL* independent pathway, has scarce data in CML. Present study investigate the association between TGFβ-Smad pathway and CML.

**Materials and Methods: **Sixty-four CML patients and age matched healthy controls (n=63) were enrolled in this study. Patients were segregated into responder and resistant groups depending on their response to Imatinib mesylate (IM). *TGFβ1* serum levels were evaluated by ELISA and transcript levels of *TGFβ1* receptors, *SMAD4* and *SMAD7* were evaluated by Real-Time PCR. Sequencing of exons and exon-intron boundaries of study genes was performed using Next Generation Sequencing (NGS) in 20 CML patients. Statistical analysis was performed using SPSS version 16.0.

**Results:** *TGFβ1* serum levels were significantly elevated (*p *= 0.02) and *TGFβR2* and *SMAD4* were significantly down-regulated (*p *= 0.012 and *p *= 0.043 respectively) in the patients. c.69A>G in *TGFβ1*, c.1024+24G>A in *TGFβR1* and g.46474746C>T in *SMAD7* were the most important genetic variants observed with their presence in 10/20, 8/20 and 7/20 patients respectively. In addition, *TGFβR1* transcript levels were reduced in CML patients with c.69A>G mutation. None of the genes differed significantly in terms of expression or genetic variants between responder and resistant patient groups.

**Conclusion:** Our findings demonstrate the role of differential expression and genetic variants of TGFβ-Smad pathway in CML. Decreased *TGFβR2* and *SMAD4* levels observed in the present study may be responsible for reduced tumor suppressive effects of this pathway in CML.

## Introduction

 Chronic Myeloid Leukemia (CML) is a hematological disorder, caused due to the transformation of pluripotent stem cells from progenitors to malignant cells. The disease process of CML initiates with the formation of Philadelphia chromosome,a unique chromosome created by reciprocal chromosomal translocation of *BCR* gene from chromosome 9 to chromosome 22^1^. Expression of this fusion gene is responsible for molecular alterations which increases malignant myelopoiesis and alter normal blood cell production^2^.Imatinibmesylate (IM) is the frontline therapy for CML. It is a selective tyrosine kinase inhibitor (TKI) targeted against *BCR*-*ABL*, which successfully halts disease progression towards blast phase in most of the patients^3^. However, a significant proportion of patients (approximately 20%-25%) show primary or secondary resistance to IM therapy. Hence,it is important to assess the role of other potential therapeutic targets in CML. Several molecular pathways, including *BCR *- *ABL* dependent and independent, are under investigation for their role in IM resistance^4^^,^^5^. Transforming growth factorβ (TGFβ) -Smad pathway is a multifunctional molecular pathway, which regulates different cellular activities like apoptosis, metamorphosis, differentiation, proliferation, angiogenesis, remodeling of extra-cellular matrix, etc^6^. *TGFβ1*, a pleiotropic cytokine, binds to receptor *TGFβR2*, which in turn recruits *TGFβR1*^7^. Activated *TGFβR2*/*TGFβR1* complex phosphorylates the receptor-Smads (R-Smads), *Smad2 *and *Smad3*. R-Smads phosphorylate and form a higher order complex with common-Smad (Co-Smad), *SMAD4*, and translocates to the nucleus for expression of target genes. Inhibitory Smads (I-Smads), *Smad6* and *Smad7,* prevent phosphorylation of R-Smads by *TGFβR1*. *Smad6* is known to play its role in BMP-Smad pathway, whereas *SMAD7* is involved in TGFβ-Smad pathway^8^^, ^^9^^, ^^10^. 

Several evidence demonstrate association of TGFβ-Smad pathway with cancer^11^. Loss of functional mutations in *TGFβR2* or decreased expression of the receptor due to epigenetic changes have been reported in various cancers, including colorectal, gastric, ampullary carcinomas, gliomas, etc^12^^, ^^13^^, ^^14^. Further, in human oral carcinoma, metastatic cells show significantly reduced *TGFβR2* levels as compared to primary tumors ^15^. Low expression of *SMAD4* has been reported in various cancers and is linked with better prognosis^16^^, ^^17^. In hematological malignancies including AML and T-cell lymphomas, low levels of *SMAD4* have been documented ^18^^, ^^19^. 

Association of TGFβ-Smad pathway with CML is not well established and needs to be explored. This encouraged us to study the expression and genetic variant in genes of this important pathway in CML. We examined the serum levels of ligand *TGFβ1* and expression of its receptors *TGFβR1* and *TGFβR2*, Co-Smad (*SMAD4*) and I-Smad (*SMAD7*) in CML patients, Association between genetic mutations in study genes and CML was also analyzed in a subset of CML patients. 

## MATERIALS AND METHODS

**Subjects**

All patients (>18 years) diagnosed with CML were prospectively enrolled for a period of two years (October 2013-October 2015) at Department of Medical Oncology, Sir Ganga Ram Hospital, Delhi, India. Diagnosis was confirmed by reverse transcription polymerase chain reaction (RT-PCR) for *BCR*-*ABL* fusion gene and fluorescent in situ hybridization (FISH) for translocation (9; 22). Enrolled patients were segregated as responders and resistant as per European Leukemia Net, 2013 (ELN, 2013) recommendations. At the time of enrollment, patients’ clinical and demographic data were obtained (Table 1). Healthy subjects with no known history of malignancy and above 18 years of age were enrolled as age-matched controls. The study was reviewed and approved by the Ethics Committee of Sir Ganga Ram Hospital (EC No.: EC/11/12/439). Informed consent was signed and submitted by all subjects at the time of enrollment. 

Peripheral blood sample in EDTA vials and plain vials (for serum) was obtained from both patients and controls. Serum was collected to compare *TGFβ1* levels and stored at -80^o^C. Peripheral blood RNA and DNA were immediately extracted (Nucleospin RNA#740200 and Nucleospin DNA#740951 Macheley-Nager, Duren, Germany) and stored at -80^o^C for further use.

**Expression levels**

Estimation of serum TGFβ1 levels

The *TGFβ1* serum levels between patients and healthy controls were measured using TGFB1 sandwich ELISA (DRG Instruments GmbH#EIA1864, Marburg, Germany). Briefly, standard and serum samples were diluted in assay buffer, acidified with hydrochloric acid (HCl), and then neutralized samples were added to the antibody coated microtiter wells. The unbound serum was washed and a biotinylated anti TGFβ1 IgG antibody was added, followed by incubation with streptavidin-HRP Enzyme complex, and then unbound conjugate was washed off. Substrate solution was added, and absorbance (OD) of each well at 450 nm was taken with a microtiter plate reader (Infinite M200). The intensity of developed color in standard was considered as proportional concentration and the *TGFβ1 *serum levels were calculated using standard curve in the patients and control samples. Median was calculated to evaluate the relative difference in the *TGFβ1 *levels of patients and controls.

Examining transcript levels of the candidate genes 

Total 1µg of RNA was converted to cDNA in a 20 µl of reaction having random primers, dNTP’s, reaction buffer and reverse transcriptase enzyme using high capacity cDNA Reverse Transcription Kit (Applied Biosystem#4368814, Vilnius, Lithuania). The transcript level of *TGFβR1*, *TGFβR2*, *SMAD4* and *SMAD7* was examined by Real-Time PCR (Stratagene Mx3005P) using SYBRgreen chemistry (Applied Biosystem#43855612, Vilnius, Lithuania). Briefly, 25 ng of cDNA was used to prepare 10 µl of reaction containing respective primers and SYBRgreen. *ACTB (β-actin)* was used as an endogenous gene. The raw data was analyzed manually by 2^-∆Ct^ method and the median of 2^-∆Ct ^was compared between patients and controls. **Identification of genetic variants**

Next Generation Sequencing 

A targeted panel with probes covering all coding exons and essential splice sites for *TGFβ1*, *TGFβR1*, *TGFβR2*, *SMAD4* and *SMAD7* genes was used for sequencing these samples using Illumina’sTruSight technology (Illumina, San Diego, USA). Genomic DNA isolated from blood was quantified using Qubit (Thermo Fisher Scientific, Waltham, USA) and 50 ng was taken for library preparation. Briefly, DNA was subjected to fragmentation and tagged with adaptors, platform-specific tags and barcodes to prepare the DNA sequencing libraries. The tagged and amplified sample libraries were checked for quality using BioAnalyzer (Agilent2100, Santaclaraca, USA) and quantified using Qubit. 500 ng of each library was pooled with other samples and hybridized to biotinylated probes. The hybridized target DNA fragments were pulled down using streptavidin beads. Two successive enrichment steps were performed to optimize the pull down of the regions of interest. Target libraries were amplified using limited PCR steps and loaded for sequencing on the MiSeq (Illumina, San Diego, USA) to obtain ~3 GB per sample. 

**Sequence analysis**

The trimmed FASTQ files were generated using MiSeq Reporter from Illumina. The reads were aligned against the whole genome build hg19 using STRAND® NGS V2.1.6 (Strand Life Sciences Pvt. Ltd., Bangalore, India). Five base-pairs from the 3' end of the reads were trimmed, as were 3' end bases with quality below 10. Reads which had length less than 25 bp after trimming were not considered for alignment. A maximum of 5 matches of alignment score at least 90% were computed. Reads that failed quality control, reads with average quality less than 20, reads with ambiguous characters were all filtered out. The STRAND® NGS variant caller was used to detect variants at locations in the target regions covered by a minimum of 10 reads with at least 2 variant reads. Variants with a decibel score of at least 50 were reported. 

**Interpretation**

Interpretation of the variant data was done using the Strand Omics software, V1.9. Strand Omics is a clinical genomics interpretation and reporting platform developed at Strand Life Sciences, Bangalore. The variant annotation engine includes algorithms to identify variant impact on gene using both public content (HGMD, ClinVar, OMIM, HPO, links to dbSNP, 1000 Genomes, Exome Variant Server, and built-in algorithms SIFT, PolyPhen HVAR/HDIV, Mutation Taster and LRT) and proprietary content (curated variant records). Interpretation interface in Strand Omics allows quick filtering and evaluation of variants along with capture of justification for inclusion/ exclusion.

**Statistical analysis**

Statistical analyses were performed using SPSS Version 16.0 (SPSS, Chicago, USA). Continuous variables are presented as mean (range) or median [interquartile range (IQR)] as appropriate. Categorical variables are presented as absolute numbers. For two groups, comparison of parametric and non-parametric data student’s t- test and Mann-Whitney U test was used respectively. P- value <0.05 was considered as significant.

## Results

 CML patients (n=64) and age-matched healthy controls (n=63) were enrolled in the study. In our patient cohort, 47 patients were IM responder and 17 were IM resistant. Demographic and clinical features of subjects are described in Table 1. 

**Table 1:** Demographic and clinical variables of CML patients and controls enrolled in the study.

**Demographic/ Clinical Variables**	**Groups**	**CML Patients (n = 64)**	**Healthy Controls (n = 63)**	**p- value**
Sex	Male/Female	53/11	51/12	p=ns^a^(0.129)
Age (years)	Mean (Range)	41 (19-72)	40 (19-68)	p=ns^b^(0.946)
Groups	Resistant	17	N/A	
	Responders	47	N/A	

Data is presented as mean (range) or n (number of patients) as appropriate. Signiﬁcance testing was performed by

a χ2 test;

b Student t-test and p<0.05 was considered signiﬁcant.

**Serum level of** ***TGFβ1***

**FIGURE 1:**
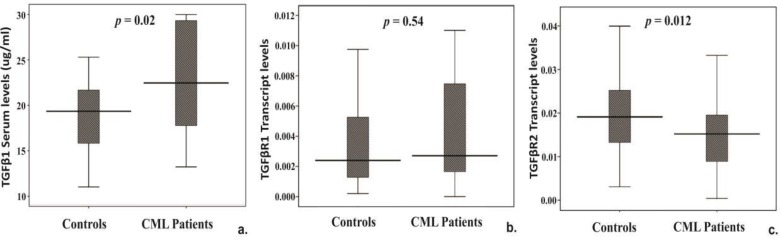
Box-plot representation of (a) TGFβ1 serum levels in CML patients (n=25) and healthy controls (n=26). Transcript levels of (b) TGFβR1 and (c) TGFβR2 in CML patient with healthy controls. In the graph, central line represents median, boxes represent 25th-75th percentile and whiskers indicate minimum and maximum values. P- values <0.05 considered significant.

*TGFβ1 *serum levels were compared between 25 patients and 26 healthy controls. We observed a significant elevation of *TGFβ1 *serum levels in patients [median (IQR) = 22.5µg/ml (17.5 - 29.4)] compared to healthy controls [median (IQR) = 19.3µg/ml (14.9 - 25.3) (*p*=0.020)] (Fig 1a). 

**Transcript level of** ***TGFβ1*** **receptors,** ***SMAD4*** **and** ***SMAD7***

**FIGURE 2:**
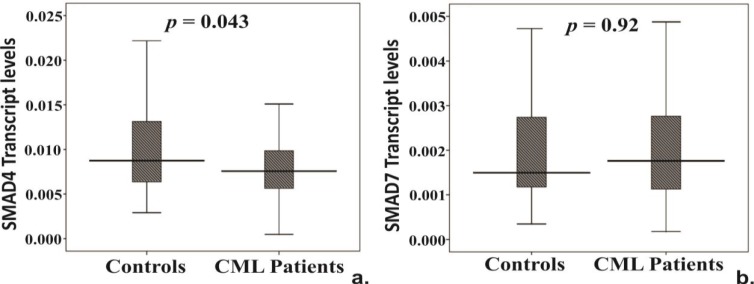
Transcript levels comparison between CML patients and healthy controls in (a) SMAD4 and (b) SMAD7. In the graph, central line represents median, boxes represent 25th-75th percentile, and whiskers indicate minimum and maximum values. p values <0.05 considered significant.

Transcript levels of *TGFβ1* receptors (*TGFβR1* and *TGFβR2*), SMAD4 and SMAD7 were examined in CML patients and healthy controls. Interestingly, *TGFβR2* was significantly down-regulated in patients [Patient median (IQR) = 0.015 (0.08-0.09), Controls median (IQR) = 0.019 (0.013-0.025), *p *= 0.012] (Fig 1c); however, we did not observed any significant difference in *TGFβR1 *levels (Fig 1b).Transcript levels of *SMAD4, a *key downstream gene of *TGFβR2, *were significantly reduced in patients [median (IQR) = 0.0075 (0.005-0.009)] compared to controls [median (IQR) = 0.0087 (0.0063-0.0132)] (*p *= 0.043) (Fig. 2a),but no change was observed in the inhibitory *SMAD7* (Fig.2b).Transcript levels of all the five genes were also analyzed between IM responsive and resistant patient groups. However, no major difference in the expression of the candidate genes was observed between the groups. 

**Identification of genetic variants in the candidate genes of TGFβ-SMAD signaling pathway**

**FIGURE 3:**
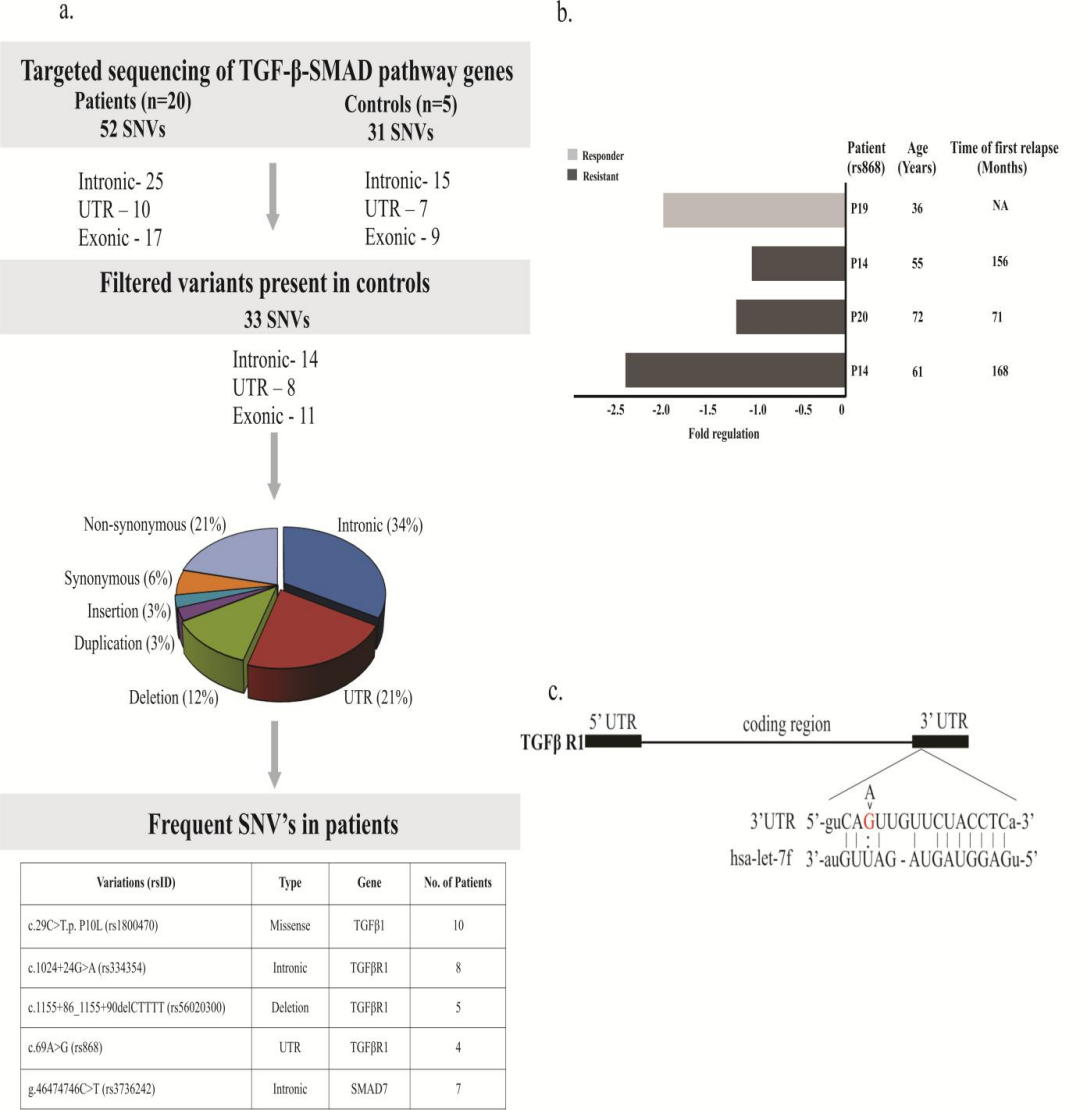
(a) Data analysis work flow of customized exome sequencing panel of TGFβ-Smad pathway genes. The pie chart represents the distribution of genetic variants. Table at the bottom represents key genetic variants. (b) Fold regulation of TGFβR1 transcript in the patients harboring mutation c.69A>G (rs868) with patients details (c) Schematic of hsa-let7f/miR98 binding site in 3’UTR of human TGFβR1 (NM_001130916) in 5’-3’ direction aligned with hsa-let7f/miR98 sequence. Fractured line between G in the UTR sequence and U in the miR sequence represents the site of mutations (A>G).

We sequenced exons and exon-intron boundaries of *TGFβ1*, *TGFβR1*, *TGFβR2*, *SMAD4* and *SMAD7* genes in a cohort of 20 patients and 5 healthy controls. Collectively, 52 variants were identified, and 33 variants were left in patients after filtering common variants. In these genetic variants, 14 were intronic, 11 were coding variants and 8 were in the untranslated region (UTR). Among intronic mutations, 11 were single-base substitution, 2 were deletion and 1 was insertion. However, 7 non-synonymous, 2 synonymous and 2 deletion variants were discovered in the coding region (Fig.3a).

In *TGFβ1* gene, 10 genetic variants were identified (Table 2). Most of the variations were confined to small number of patients, but a non-synonymous variant in exon 1(c.29C>T, p.P10L.rs1800470) was present in 50% of patients examined. In *TGFβR1*, 5 genetic variants were identified, but an intronic variant (c.1024+24G>A, rs334354) was present in 40% of patients. Another variant (c.69A>G, rs868) observed in UTR was found identified in 4 patients.

In *TGFβR2*, 5 genetic variants were observed, but all the variations were present in ≤3 patients (Table 2). Seven genetic variants were discovered in *SMAD4,* 3 of them were intronic, 3 in UTR region and 1 variant was in exon 2. None of the variants were present in more than one patient, making it impossible to relate them to change in *SMAD4* transcript levels or to associate with the disease (Table 2). Six variants were observed in *SMAD7*. Among the variants, an intronic variant g.46474746C>T (rs3736242) was present in 8 out of 20 CML patients (Table 2). 

## Discussion

 CML is diagnosed by the presence of *BCR-ABL* gene and treated by Imatinibmesylate (TKI) in first- line setting. Alterations in *BCR-ABL* dependent and independent pathways are the cause of resistance to IM in CML ^20^^.^ TGFβ-Smad is one of the key *BCR-ABL* independent pathways, which has been extensively studied in normal and abnormal hematopoiesis^21^. Alterations in this pathway have been implicated in lymphocytic^22^ and myeloid leukemias^23^, but its role in CML is not well established so far. TGFβ-Smad signaling is known to increase the hyper-responsiveness of CML cells,leading to better response through *BCR-ABL* inhibition^24^. Although the pathway inhibits the activation of *AKT*, which is a downstream component of *BCR-ABL* pathway, it also leads to release of inhibitory sequestration of *FOXO, *which promotes quiescence in CML stem cells, and ultimately results in TKI resistance^25^^, ^^26^. Present study attempted to explore more direct links between alterations in TGFβ-Smad signaling pathway and CML patients. 

*TGFβ1,* cytokine is a strong inhibitor of progenitor cell growth and differentiation, and its autocrine production maintains immature hematopoietic progenitor cells in quiescent state. Significant elevation was observed in *TGFβ1* serum levels in CML patient group as compared to controls group.

Higher levels of *TGFβ1* have been observed in hematological malignancies^27^, and solid tumors ^28^^, ^^29^which is consistent with our findings.

**Table2:** Description of genetic variants observed in CML patients

**Genetic Variants**	**rsID**	**Variant Type**	**Intron/ Exon / UTR**	**N0. Of Patients**
*TGFβ1* (NM_000660)				
c.250A>T. p. T84S	-	Missense	Exon1	1
c.74G>C. p. R25P	rs1800471	Missense	Exon1	3
c.29C>T. p. P10L	rs1800470	Missense	Exon1	10
c.635-93_635-92insA	-	Insertion	Intron3	1
c.861-20C>T	-	Intronic	Intron5	4
c.1014G>C. p. K338N	-	Missense	Exon6	1
c.58G>C	-	UTR	3’UTR	1
c.52G>C	-	UTR	3’UTR	2
c.47G>C	-	UTR	3’UTR	4
c.26dupC	-	Duplication	3’UTR	1
*TGFβR1* (NM_001130916)				
c.574+39A>G	rs11568778	Intronic	Intron3	5
c.1024+24G>A	rs334354	Intronic	Intron6	8
c.1155+86_1155+90delCTTTT	rs56020300	Deletion	Intron7	5
c.1156-15delT	-	Deletion	Intron7	2
c.69A>G	rs868	UTR	3’UTR	4
*TGFβR2* (NM_001024847)				
c.169+99T>C	rs117998227	Intronic	Intron2	2
c.458delA.p.K153SfsTer35	rs79375991	Deletion	Exon4	3
c.1242C>T	rs2228048	Synonymous	Exon5	2
c.1156-15delT	-	Intronic	Intron7	1
c.1599+62A>G	rs192590842	Intronic	Intron7	1
*SMAD4* (NM_005359c)				
c.604G>T. p. A202S	-	Missense	Exon5	1
c.905-52A>G	rs948589	Intronic	Intron7	1
c.955+58C>T	rs948588	Intronic	Intron8	1
c.1448-49G>C	rs375313666	Intronic	Intron11	1
c.7T>A	-	UTR	3’UTR	1
c.1G>T	-	UTR	3’UTR	1
c.12G>C	-	UTR	3’UTR	1
*SMAD7* (NM_001190823)				
c.179-106C>T	rs76886865	Intronic	Intron1	1
c.608C>T.p.T203M	-	Missense	Exon2	1
c.393C>G. p. C131W	-	Missense	Exon2	1
c.330C>T. p. L110L	rs3809922	Synonymous	Exon2	1
g.46474795delG	-	Deletion	Exon2	3
g.46474746C>T	rs3736242	Intronic	Intron2	7

Circulating *TGFβ1* protein concentration levels were associated with mutation c.29C>T (rs1800470) in exon 1 of *TGFβ1* gene^30^^,^^31^. We discovered this mutation in 50% of patients of the cohort selected for sequencing. Interestingly, elevated *TGFβ1 *levels were observed in 3 patients (serum levels available) harboring this mutation, but due to small sample size, the correlation between serum levels and 29C>T mutation could not be clearly demonstrated in our study. It lies in the conserved region and expected to be damaging by in silico analysis. It is speculated that Proline to Leucine (P10L) change modifies the peptide polarity, leading to change in protein transport rate^32^. We are the first to report this mutation in CML to the best of our knowledge.

A recent in-vitro study suggests that BCR-*ABL* expression enhance *TGFβ1* levels and TGFβ signaling activity in CML cell lines^33^, which prompted us to inquire whether increased serum levels in our cohort are also leading to increased signaling activity. Evaluation of *TGFβ1* receptor transcript levels showed significantly reduced *TGFβR2 *expression, which probably hamper tumor suppressive effect of *TGFβ1* in CML patients. The finding was similar with an earlier study, where decreased *TGFβR2* levels were reported in CML patients compared to healthy individuals^34^. The attempt to correlate the reduced transcript levels with genetic mutations in our cohort couldnot reveal significant observation as no mutation was present in enough number of patients to suggest such association. However, some important genetic variants were observed in *TGFβR1* gene. Genetic variant, c.69A>G (rs868), present in 3’ UTR of *TGFβR1*, was found in 20% (4/20) patients. *In silico* analysis of this variant shows the mutation site to be the target for miRNA Let7f/miRNA98 (Fig 3c). The Let7f/miR98 family is known to reduce *TGFβR1* expression during embryogenesis and mutation in the binding region of this miRNA further reduces expression of gene^35^. Analysis of transcript levels in 4 patients having this mutation demonstrated reduced *TGFBR1* transcript level; however, no significant change in expression was observed in overall patient group (Fig. 3b). Out of these 4 patients, 3 were IM resistant and showed first relapse after consuming standard dose (400mg O.D.) of imatinib mesylate for 6 years or more. The fourth patient harboring this variant completed sixth year of standard IM treatment and was a good responder till sample collection (Fig 3b). Correlation of this finding with clinico-demographic characteristics signaled towards the probable role of this variant in late relapse. Though this claim requires concrete evidence in a larger cohort, the hint is worth paying attention. Another variant, c.1024+24G>A (rs334354) in intron 6 of *TGFβR1, *discovered in 40% (8/20) of our patients is an established genetic marker for increased susceptibility for cancer^27^^, ^^36^. 

*Smad4* is a key component of TGFβ-Smad signaling and an important marker in colorectal cancer (CRC). Down-regulation of SMAD4 in CRC, due to increased miRNA, is responsible for its controlled expression^37^_._ *Smad4* deficiency has been observed in malignancies of diverse origins like oral epithelial cells, keratinocytes, mammary cells, bile duct, odntoblasts^38^^,^^43^ and leukemic cells of Chinese patients^44^. Our study findings also revealed significantly reduced SMAD4 levels along with low TGFBR2 levels. *SMAD4* is essential for the formation of heterologous complex with SMAD2 and SMAD3 and its translocation into the nucleus for expression of target genes. Its low expression can be another potential reason for containment of this tumor suppressor pathway. 

## CONCLUSION

 In conclusion, CML patients have elevated *TGFβ1* serum levels and c.29C>T is the major genetic variant among *TGFβ1* gene mutations. Lower transcript levels of *TGFβR2* can be the possible reason of decreased signaling activity that abolishes the tumor suppressor effect of the increased *TGFB1* levels. Though no significant change in the transcript levels of *TGFBR1* was observed in patients compared to control, *TGFBR1* levels were reduced in the patients with c.69A>G variant. We also reported low levels of *SMAD4* in CML. Previous studies have also reported similar findings in various other types of cancer, including hematological malignancies such as acute myeloid leukemia and T-cell lymphoma^17^^, ^^18^.

Although our results are encouraging, but detailed research on TGFβ - SMAD signaling pathway in different CML models is required to substantiate our findings. 
